# Oxidative functionalization of carbon nanotubes for enhanced adsorptive removal of phthalate esters in environmental systems

**DOI:** 10.1039/d6na00257a

**Published:** 2026-07-27

**Authors:** Kaviarasu G, Selva Kumar T, Raju Sasikumar, Ravikumar Rajarathinam, Sheena Haorongbam, Senthilkumar, Amit K. Jaiswal

**Affiliations:** a Department of Agribusiness Management and Food Technology, North-Eastern Hill University, Tura Campus Chasingre, Tura – 794002 West Garo Hills Meghalaya India sashibiofoodster@gmail.com; b Vel Tech Rangarajan Dr Sagunthala R&D Institute of Science and Technology Chennai 600062 India; c Department of Food Technology, Kongu Engineering College Erode Tamil Nadu India; d School of Food Science and Environmental Health, Technological University Dublin – City Campus Dublin Ireland amit.jaiswal@tudublin.ie; e Centre for Sustainable Packaging and Bioproducts (CSPB), Technological University Dublin – City Campus Dublin Ireland

## Abstract

Phthalic acid esters (PAEs) are high-volume manufacturing organic materials used in plasticisers to increase the elasticity of the core polymer. They are reported to spread into the surroundings from plastic products and are now a global environmental contaminant. Several environmental sources, particularly water, have been examined for phthalate contaminant levels. These substances are discharged directly into the environment and cannot be chemically bound. The presence of PAEs in different ecological environments is a major concern since they are endocrine disruptors and can interact with hormones, which can lead to problems with development and reproduction. Earlier studies have explored the occurrence, fate, and concentration of phthalates. This review discusses how different oxidative techniques employed for modifying carbon nanotubes (CNTs) can improve their ability to remove PAEs by changing their surface chemistry. Several oxidation approaches introduce oxygenated functional groups on the CNT surface, which increase their specific surface area, making them suitable for adsorbing contaminants like phthalate esters. The adsorption capacity was found to be controlled by the nanotubes' specific surface areas, which varied with their outer diameters. Because of the π–π interaction between the graphitic surface of the nanotubes and the benzene ring of the phthalates, as well as hydrogen bonds, charge-assisted hydrogen bonding, van der Waals forces, and electrostatic interactions between PAEs and CNTs, the adsorption capacity was much higher than that of other materials. PAEs' toxicological and environmental effects have drawn global attention. The present article reviews the use of oxidised CNTs in advanced carbon materials for removing PAEs from polluted and hazardous environments.

## Abstract 2

Carbon nanotube functionalization improves phthalate ester adsorption, enabling efficient wastewater treatment and environmental protection. Image generated with AI.

## Graphical abstract

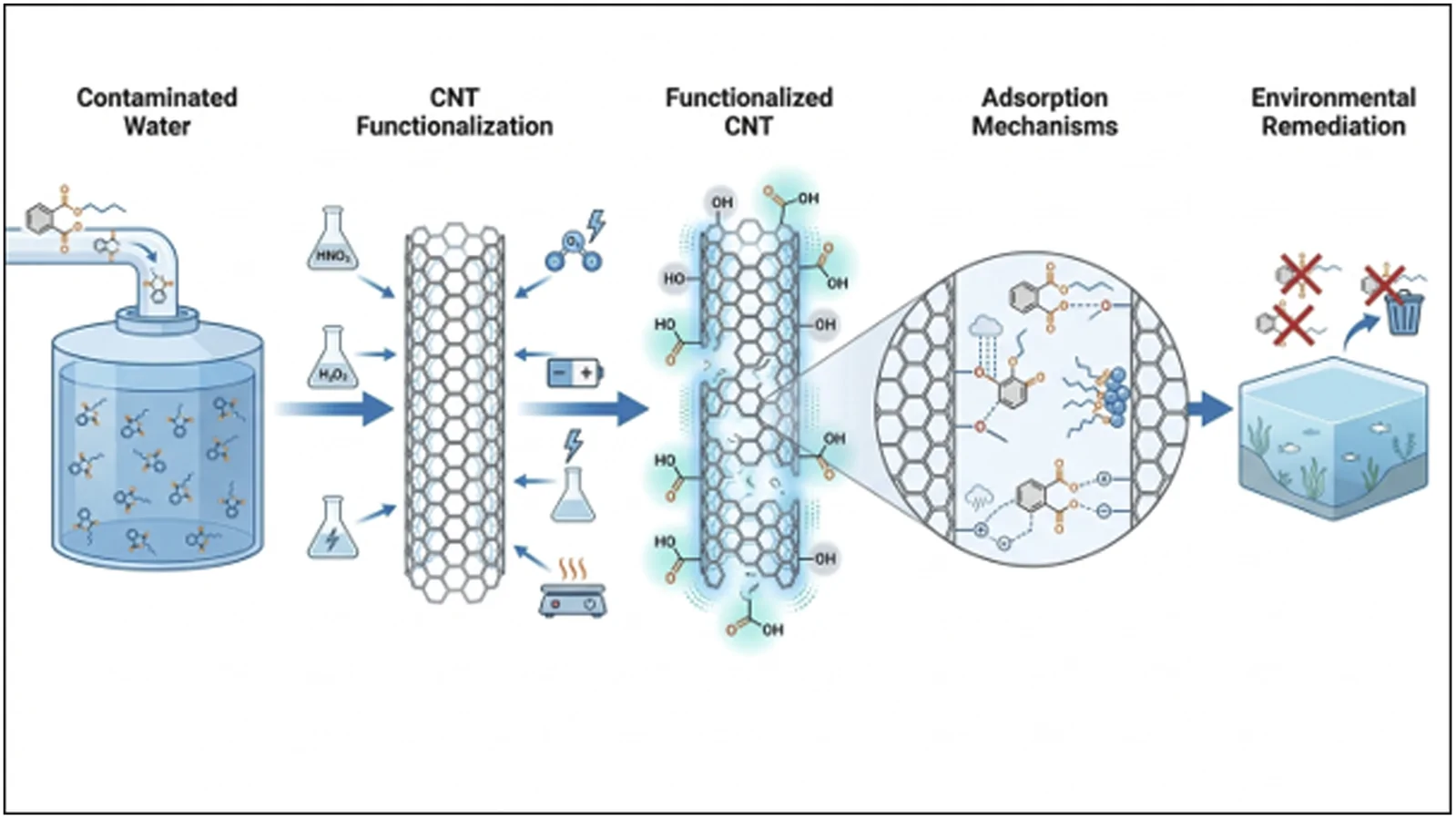

## 1 Introduction

PAEs are a group of synthetic organic compounds commonly used as plasticisers in numerous industrial and agricultural sectors, including plastics, agricultural films, construction materials, pharmaceuticals, household items, toys, electrical appliances, cosmetics, flavouring substances, and packaging materials. The demand for global plastic products has increased massively in recent years.^1^ The global use of plastic products was estimated up to 10.3 million tons by 2019. Plastic products may contain about 65% PAEs. More than 8 million tons of PAEs are produced worldwide each year. In 2016, diethylhexyl phthalate (DEHP) remained the most commonly used plasticiser, with global consumption of 3.1 million tons.^2^ In plastics, PAEs serve as “lubricants” between molecules, bonding to polyolefin-based plastics *via* van der Waals forces or hydrogen bonds while maintaining their intrinsic chemical characteristics. As a result, PAEs can rapidly leach from plastic materials into the environment. However, wastewater treatment systems could only eliminate 18% of PAEs under standard conditions. PAEs have been found in nearly every environmental matrix, including soil, water, sediment, air, and biota.^3^

In addition to their high matrix complexity and low concentrations, organic chemicals such as PAEs are toxic and carcinogenic, making their extraction problematic. Additionally, one of the most difficult and complex laboratory research project procedures has always been sample preparation.^4^ Pre-concentration is essential for detecting and measuring trace levels of contaminants, like PAEs, in a sample before any instrumental analysis. Various pre-treatment techniques are available, including liquid–liquid extraction (LLE), solid-phase extraction (SPE), accelerated solvent extraction (ASE), single-drop microextraction (SDME), liquid-phase microextraction (LPME), and solid-phase microextraction (SPME). However, these techniques may have several disadvantages, including limited effectiveness, high costs, significant energy requirements, reliance on specific chemicals, and challenges regarding sludge disposal.^5^

The exceptional adsorption ability of carbon nanotubes (CNTs) across a broad range of analytes and matrices has been demonstrated in several recent studies. Since CNTs are used as PAE sorbents, these molecular-scale pipes of graphitic carbon, which can be thought of as a cylinder-shaped graphene sheet, have the following special qualities, such as a high surface area, the capacity to create π–π interactions, high mechanical and chemical stability, and insolubility in organic solvents and water, among others. They can be further solubilized by functionalizing them or by adding surfactants, if needed, and functionalization can also make them more reactive.^6^ Defect sites are created on the graphitic network due to intense contact between oxidizing acid molecules and CNTs during treatment. To develop functional groups like hydroxyl (–OH) and carboxylic acid (–COOH) on the CNT surface, these oxidative defects are created by substituting one or more oxygen atoms for one carbon atom in the CNT lattice. Under aqueous conditions, these functional groups improve the hydrophilicity and dispersibility of CNTs. This results in CNTs with improved adsorption capacity, greater active-site accessibility, faster adsorption kinetics, and better regeneration potential during adsorption/desorption cycles.^7^ The primary aim of this review is to comprehensively investigate the oxidation of CNT-based materials, which are widely used to eliminate organic polymer contaminants, like PAEs, from the environment. The structure of PAEs and their occurrence in different environmental sources, along with their health impacts, are examined. Subsequently, the oxidation process enhances the interactions between CNTs and PAEs through mechanisms including π–π interactions, hydrophobicity, hydrogen bonding, and charge-assisted hydrogen bonding.

## 2 PAEs' structure and behaviour

PAEs, often referred to as phthalate esters, are dialkyl or alkyl aryl derivatives of 1,2-benzenedicarboxylic acid, commonly known as phthalates. Phthalic anhydride is reacted with different alcohols by Fischer esterification to form phthalate esters, which enable the production of a variety of phthalates.^8^ PAEs are hydrophobic substances with log *K*_ow_ values between 1.6 and 12, based on hydrogen-bonding and van der Waals interactions.^8^ Pollutants that are poorly decomposed in water cannot break down, while their characteristics enhance their separation into sediments and environments.^9^ The higher boiling point (230–486 °C) and lower melting point (below −25 °C) of PAEs contribute to their applicability as plasticisers and heat-transfer fluids, as well as to the length of the hydrocarbon chain. PAEs can be divided into two groups: low molecular weight (LMW) compounds, which contain C1–C4 chains and are relatively more water-soluble. High-molecular-weight (HMW) compounds have relatively low water solubility because they have long carbon chains (Table 1).^10^

**Table 1:** Basic physicochemical properties, molecular information, and common industrial and consumer applications of selected PAEs (source: PubChem database, https://pubchem.ncbi.nlm.nih.gov)

PAEs	Molecular formula	Molecular weight (g mol^−1^)	CAS registration number	Specific gravity (20 °C)	Water solubility (mg L^−1^)	Log *K*_ow_	Melting point (°C)	Applications
Dimethyl phthalate (DMP)	C_10_H_10_O_4_	194.18	131-11-3	1.19	4000	1.6	8	Solvent and insect repellent
Diethyl phthalate (DEP)	C_12_H_14_O_4_	222.24	84-66-2	1.12	1080	2.5	−50	Cosmetics and fragrances
Dipropyl phthalate (DPrP)	C_14_H_18_O_4_	250.29	131-16-8	1.09	140	3.0	−20	Plasticiser and coatings
Diisopropyl phthalate (DiPrP)	C_14_H_18_O_4_	250.29	605-45-8	1.07	60	3.2	−60	Plasticizer
Dibutyl phthalate (DBP)	C_16_H_22_O_4_	278.34	84-74-2	1.05	13	4.5	−35	Plasticiser and adhesives
Diisobutyl phthalate (DiBP)	C_16_H_22_O_4_	278.34	84-69-5	1.04	11	4.1	−64	Plasticiser and inks
Dipentyl phthalate (DPP)	C_18_H_26_O_4_	306.40	131-18-0	1.02	1.5	5.0	−20	Plasticizer
Monobenzyl butyl phthalate (MBBP)	C_18_H_18_O_4_	298.33	27 613-35-0	1.20	120	3.3	80	Metabolite/derivative
Benzyl butyl phthalate (BBP)	C_19_H_20_O_4_	312.36	85-68-7	1.11	2.7	4.8	−35	Vinyl flooring and PVC
Dicyclohexyl phthalate (DCHP)	C_20_H_26_O_4_	330.42	84-61-7	1.11	0.3	5.2	60	Specialty plasticizer
Dihexyl phthalate (DHP)	C_20_H_30_O_4_	334.45	84-75-3	1.02	0.5	6.0	−10	Plasticizer
Di(2-ethylhexyl) phthalate (DEHP)	C_24_H_38_O_4_	390.56	117-81-7	0.99	0.003	7.5	−50	PVC and food-contact plastics
Di-*n*-octyl phthalate (DnOP)	C_24_H_38_O_4_	390.56	117-84-0	0.98	0.01	8.0	−25	Cable insulation
Diisononyl phthalate (DINP)	C_26_H_42_O_4_	418.62	28 553-12-0	0.97	0.002	8.8	−40	Flexible plastics
Diisodecyl phthalate (DIDP)	C_28_H_46_O_4_	446.68	26 761-40-0	0.97	<0.001	9.5	−45	Automotive plastics
Diundecyl phthalate (DUP)	C_3_H_50_O_4_	474.72	3648-20-2	0.96	<0.001	10.0	−20	Heavy-duty plasticizer
Ditridecyl phthalate (DTDP)	C_32_H_54_O_4_	502.78	119-06-2	0.95	<0.001	10.6	−5	Industrial PVC
Bis(2-hydroxyethyl) phthalate	C_12_H_14_O_6_	254.24	605-45-8	1.30	5000	1.3	110	Degradation product

### 2.1 Occurrence of PAEs

PAEs are the primary compounds in plastic materials, comprising 10–60% of the product's weight. PAEs are readily released into the environment during manufacturing, transportation, abrasion, disposal, prolonged release from PVC plastics, and direct or indirect wastewater discharges.^11,12^ Each year, PVC is produced in the millions of tons, with soft PVC accounting for approximately 87% of all PAEs. Polyethylene terephthalate (PET) is a secondary source of PAE contamination in the environment. Terephthalic acid or its methyl ester, catalyzed by antimony oxide, reacts with ethylene glycol to create PET. Maintaining appropriate temperature and humidity levels during PET production is crucial. High temperatures, elevated moisture levels, and extended exposure to moisture lead to hydrolytic and thermal breakdown of the polymer chains. This breakdown damages the plastic and produces by-products such as acetaldehyde and oligomers, while also allowing phthalate plasticizers to seep into adjacent materials.^13^

The primary toxic effect of PAEs in the environment is the slow release of phthalates from plasticizers and other materials during weathering. Pollutants resist degradation by environmental microbes and accumulate in natural waters, resulting in a widespread distribution across water bodies.^14^ PAEs are present in a broad range of concentrations (0.1 × 10^−3^ to 3.2 × 10^3^ µg L^−1^) in aquatic sources.^15^ PAE levels in tap water can be influenced by a number of variables, including the water's flow distance, the materials used in the pipes, the length of the transit, and the usage of plastic packaging, all of which can contaminate drinking water. The water treatment technologies implemented at treatment plants are primarily designed to eliminate microorganisms and heavy metals present in the water.

In sludge treatment, anaerobic digestion and dehydration are often used to combine, concentrate, and stabilise sludge generated by the aforementioned procedures.^16^ After anaerobic digestion, the nitrogen in the supernatant is selectively removed using anammox, a new nitrogen removal technique. The efficiency of removing PAEs tends to decline during tertiary treatment processes, which include flocculation or coagulation, sedimentation, and disinfection. Conventional wastewater treatment techniques, such as CAS, oxidation ponds (OP), trickling filters (TF), and sequencing batch reactors (SBR), are not adept at eliminating low concentrations of PAEs. To mitigate human exposure to PAEs through normal water consumption, it is clear from the previous discussion that there is an urgent necessity to improve residential water treatment. Additionally, it is crucial to carefully evaluate the use of PAEs in the production and manufacturing of plastic packaging.^17^

### 2.2 Case studies of PAE contamination in aquatic systems

Concentrations of PAEs show considerable variation depending on the season and region and are influenced by hydrological, industrial, and climatic conditions. Research conducted in the Yangtze River estuary and the adjacent East China Sea reveals that 16 PAEs display notable seasonal and geographical differences in both seawater and sediment, primarily a result of river runoff and industrial discharge. Higher levels of DBP and DiBP are found in seawater, while DEHP tends to accumulate in sediments because of its strong hydrophobic nature. In contrast, DMP and DEP are generally found in low or negligible concentrations in both water and sediment, and DEHP in sediment typically poses minimal risks to sensitive species.^18^ The Kaveri River serves as a vital source of drinking water for the Western Ghats region of India, especially in the states of Karnataka and Tamil Nadu. Selvaraj *et al.*^19^ carried out research on the concentration of PAEs in the Kaveri River, where they discovered that 17 PAE samples exhibited high levels. Their research revealed the extensive presence of DEP, with DMP, BBP, and DEHP detected in 92% of the samples, while DBP and DNOP were found in 67%. The highest levels of DEHP (822 ng L^−1^) and BBP (145 ng L^−1^) were found in Erode, with Bhavani also showing elevated amounts of DEHP, BBP, and DEP, likely due to intensive industrial activity in these textile, rubber, paper and manufacturing hubs. Ten prevalent PAEs were examined for contamination levels and distribution patterns in several kinds of water samples taken from the Hanoi metropolitan region in Vietnam. Among PAEs, di-(2-ethylhexyl) phthalate (DEHP) made up a significant percentage of total concentrations (45%) in wastewater, followed by dibutyl phthalate (DBP, 9.53%) and diisobutyl phthalate (DiBP, 10.3%).^20^

The results demonstrate that PAEs' industrial activity, wastewater treatment techniques, and climate dynamics all influence PAEs' dispersion. The need for sophisticated adsorptive materials is underscored by PAEs' resistance to traditional treatments and their widespread presence across many regions. Table 2 displays the various concentrations of PAEs found in different regions around the world. The need for improved adsorptive materials is underscored by PAEs' resistance to traditional treatments and their widespread presence across many geographic locations.

**Table 2:** Overview of the environmental occurrence of PAEs and their associated ecological and human health effects

Place/region	Source	PAE name	Concentration	Reported/associated side effects	Ref.
Vietnam	Bottled water	DBP, DiBP, BzBP, and DEHP	1640–15,700 ng L^−1^	Endocrine disruption, reproductive toxicity, and hormonal imbalance	21
Vietnam	Wastewater	DEHP, DiBP, and DBP	20 700–405 000 ng L^−1^	Endocrine disruption, reproductive toxicity, and potential carcinogenic risk	21
China	River water	DMP, DEP, DIBP, DBP, DEHP, and DNOP	2.65–39.31 µg L^−1^	Medium to high environmental risk; DBP exceeded the surface water standard	22
China	Suspended particulate matter (SPM)	DMP, DEP, DIBP, DBP, DEHP, and DNOP	1.97–34.10 µg g^−1^	High ecological risk and bioaccumulation potential	22
China	Sediment	DMP, DEP, DIBP, DBP, DEHP, and DNOP	0.93–34.70 µg g^−1^	High environmental risk, particularly from DIBP and DEHP	22
Russia	Lake water	DBP, DEHP, and DnOP	2.30–17.34 µg L^−1^	Ecological risk to sensitive aquatic organisms	23
North China	Agricultural soil	DEHP, DBP, and DMP	191–457 ng g^−1^	Potential ecological and human health risk	24
Lithuania	Wastewater	DMP, DEP, DPP, DBP, DiBP, DCHP, and DEHP	≤159 000 ng L^−1^ (DEHP)	Endocrine disruption and ecological risk	25
Poland	Wastewater	DMP, DEP, DBP, and DEHP	≤210 000 ng L^−1^ (DMP)	Endocrine disruption and ecological risk	25
Denmark	Wastewater	DEP, DBP, and DEHP	≤1200 ng L^−1^ (DEHP)	Reduced risk due to effective management	25
Lithuania	Sediment/sludge	DMP, DEP, DPP, DBP, DiBP, DCHP, and DEHP	≤95 000 ng g^−1^	Long-term ecological risk	25
Denmark	Sediment/sludge	DEP, DBP, and DEHP	≤6600 ng g^−1^	Lower ecological risk	25
China	Agricultural soil (reclaimed-water irrigated)	DMP, DBP, and DEHP	2790–5340 ng g^−1^	Potential ecological risk and soil contamination concern	26
India	River water & drinking water	DEHP, DiBP, DEP, and DMP	≤3769 ng L^−1^	Estrogenic activity and ecological risk to fish due to DEHP	27
India	Drinking water & food	DEP, DEHP, and DBP	Water: ≤5688 ng L; food: 665 ng g^−1^	High dietary exposure and endocrine-disrupting potential	28
India	Bottled water (PET, rPET, LDPE)	DEHP and DBP	≤64 300 ng L^−1^	Migration risk exceeds the USEPA/WHO limits	29
India	Urban soil	DEP, DBP, MBBP, BHEP, and ODP	Detected (levels varied across wards)	Environmental contamination concern	30
India	Estuarine sediment	DEHP, BBP, DBP, DMP, and DEP	44–4015 ng g^−1^	DEHP & BBP exceeded risk limits and serious ecological hazard	31
India	River sediment	DMP, DEP, DBP, DEHP, and DNOP	4570–31 610 ng g^−1^	Potential ecological risk: DEHP is most frequently detected	31 and 32

## 3 Human exposure, metabolism, and toxicological impacts of PAEs

### 3.1 Human exposure pathways

**Fig. 1:**
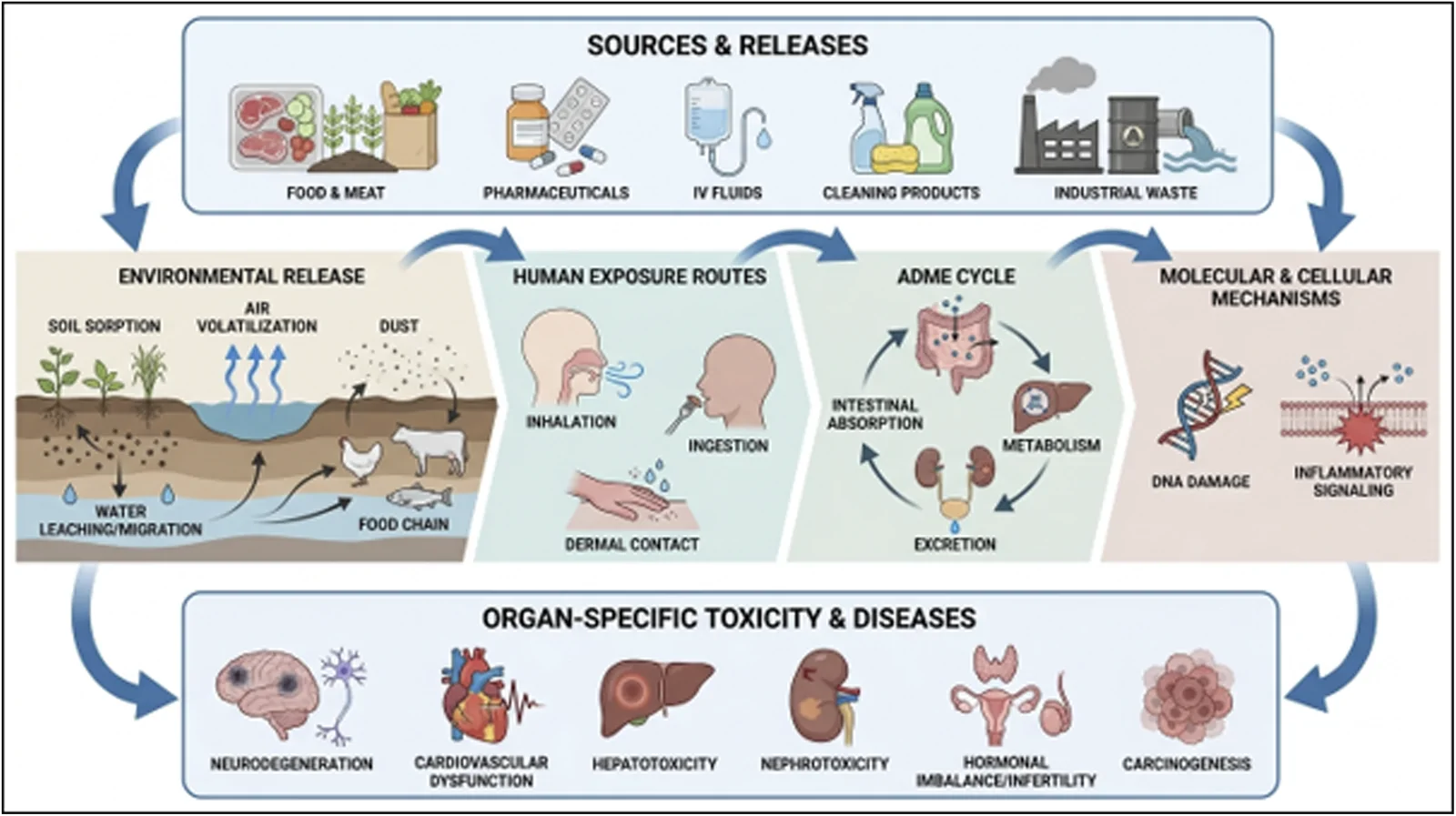
Schematic illustration of PAE sources, environmental release, human exposure and health effects. Image generated with AI.

There are several ways through which humans are exposed to PAEs, including ingestion, inhalation, skin contact, and placental transmission (Fig. 1). The exposure pathway will vary depending on the PAEs' molecular structure and weight. Oral intake is the primary pathway for exposure to high-molecular-weight PAEs. Plasticised food packaging, processing equipment, and storage containers all rapidly allow toxic PAEs to leak into consumables. While low-molecular-weight PAEs, such as DEP and DBP, are more volatile and water-soluble and are found in cosmetic products (face cream, lotion, and nail polish). They can enter the body through skin absorption or inhalation. When pregnant mothers are exposed to PAEs, they can enter the amniotic fluid and cord blood and affect the development of the fetus.^33,34^ Semi-volatile PAEs are not covalently bonded to polymeric matrices; high ambient air temperatures may promote their release from materials such as PVC flooring, increasing their levels in the air and surrounding environment. In addition to plastic and cosmetics, food and soil are also exposed to PAEs.

Due to higher usage of pesticides, PAEs are found in every part of the plant, from the roots to the stem to the seed, and they have a detrimental influence on the quality of the soil and possibly even human health.^35^

### 3.2 Toxicological impact of PAEs on human and aquatic organisms

MEHP inhibits FSH (follicle-stimulating hormone) stimulation of cAMP (cyclic Adenosine MonoPhosphate) and progesterone synthesis. Pregnenolone, a precursor to progesterone, or cAMP stimulators can ease the decline of progesterone. In contrast, MEHP inhibits the formation of estriol in response to either FSH or the nondegradable cAMP analogue, 8br-cAMP. This suggests that MEHP's effects on estradiol differed from its effects on cAMP and progesterone production.^36^ Monobutyl phthalate did not affect aromatase levels or granulosa cell estradiol production, but MEHP reduced aromatase RNA and protein levels in a dose-dependent manner.^37^ MEHP's processes appeared to target aromatase specifically, leaving transcript levels of the cholesterol side-chain cleavage enzyme (P450scc) unaffected. Additionally, the addition of 8-br-cAMP was unable to reverse the MEHP-induced decrease in aromatase levels, supporting the idea that MEHP inhibits aromatase synthesis and estradiol production without requiring FSH-stimulated cAMP activation.^38,39^

PAEs influence the male reproductive system, as demonstrated by Shaffer *et al.*,^40^ who provided the initial evidence of testicular damage caused by phthalates in experimental mice. Characteristics of the testicular effects include decreased testis weight and atrophy of the seminiferous tubules. Germ cells, mostly spermatocytes and spermatids, gradually degenerate and eventually slough off into the tubular lumen. Germ cell death, reduced testicular zinc levels, and increased zinc excretion in the urine are all associated with tubular atrophy. However, it shows that malfunctions in sertoli cells, which are unable to nourish germ cells adequately both physically and metabolically, are the cause of the changes in spermatogenesis observed following exposure to hazardous reproductive phthalates such as DEHP.^41^

In addition to reproductive harm, the recent study suggests a connection between PAE exposure and human respiratory and systemic health. Wittassek & Angerer collected urine samples from 418 teenagers (between the ages of 14 and 15) in Belgium, and they discovered that oxidative stress, assessed by urinary 8-hydroxy-2′-deoxyguanosine (8-OHdG), connects phthalate exposure to asthma. MEHP, MEOHP and MEHHP are significantly linked to increased asthma risk, with odds ratios of 1.84 and 1.94, respectively. PAEs' persistence for bioaccumulation can provide significant ecological threats to aquatic life. According to Jiang *et al.*,^43^ in a 28 day exposure study, DBP decreased acetylcholinesterase (AChE) by as much as 14.7%, increased antioxidant enzyme activity (SOD, CAT, *etc*.), and elevated malondialdehyde (MDA) and 8-hydroxy-2′-deoxyguanosine (8-OHdG) in zebrafish brain function.^42^

## 4 Carbon nanotubes as adsorbent materials

In the current generation, research is more focused on nanomaterial-based environmental remediation, specifically wastewater treatments. Among carbon-based materials, CNTs are emerging as widely used materials for removing organic pollutants, including PAEs. The graphite structure of CNTs is comparable to that of activated CNTs, which provides adsorption sites for organic pollutants.^44^ The oxidised CNTs effectively remove contaminants due to their large specific surface area, remarkable porosity, layered and hollow architectures, π-conjugated structure, numerous internal and external adsorption sites, and ease of chemical activation and functionalisation.^45,46^ PAEs, like aromatic compounds, are adsorbed onto CNTs *via* π–π interactions, though the mechanisms also include van der Waals interactions, capillary condensation, and electrostatic attractions. The narrow inner holes of CNTs adsorb small molecules and ions, with binding energies that ensure consistent retention and minimised desorption.^47^ The outer region of CNTs has a high surface area, enabling greater pollutant adsorption and supporting rapid kinetics and high adsorption efficacy. The external surface area rapidly attracts pollutants *via* electrostatic interactions, π–π stacking, and hydrophobic interactions, resulting in high adsorption of organic pollutants.^48^

### 4.1 Carbon nanotube classification

#### 4.1.1 *Types* of CNTs

CNTs were classified by structure into two categories: a one-atom-thick layer of the identical polyaromatic and monoaromatic layer composed of a hexangular display of sp^2^ hybridised carbon atoms is wrapped to form the structure of single-walled carbon nanotubes (SWCNTs). SWCNTs have a diameter of 1–2 nm, high tensile strength and sensitive electrical conductivity because of a single layer.^49,50^ Multi-walled carbon nanotubes (MWCNTs) form out of two or more graphene sheets encasing a hollow core, with outer and inner diameters ranging from 2 to 100 nm and from 1 to 3 nm, respectively. This high aspect ratio, combined with their large surface area, allows MWCNTs to efficiently adsorb contaminants, including heavy metal ions, from water and other media.^51^ MWCNTs' cylindrical layers interact with one another *via* π–π interactions, which makes the architecture less flexible and more susceptible to minor defects in the matrix.^52^ One of the most important characteristics of an adsorbent is its specific surface area, which determines the number of sites available for adsorbing molecules. One of the key factors influencing an adsorbent's capacity to absorb organic compounds is its porosity and a favourable pore size distribution. Surface functions, site density, and purity also affect the adsorption performance of CNTs.^53^ Research indicates that MWNTs are great at adsorbing pollutants due to their high pore capacity and surface area. For instance, MWNTs with 40 walls have a (Specific Surface Area) SSA of approximately 50 m^2^ g^−1^. It was noted that the SSA of bundles diminished as the number of CNTs increased, with a bundle of 7 SWNTs resulting in an SSA of 751 m^2^ g^−1^ and a bundle of 217 SWNTs reaching an SSA of 151 m^2^ g^−1^. Also, a correction factor for the SSA in bundles was established, with a value of 0.571 for 7 CNTs (Table 3).^54^

**Table 3:** BET specific surface area (SSA) changes of CNTs after functionalization

CNT type	Oxidation/activation method	SSA before functionalization (m^2^ g^−1^)	SSA after functionalization (m^2^ g^−1^)	ΔSSA (m^2^ g^−1^)	% Change	Ref.
MWCNTs	Hydrothermal oxidation	278	441	163	58.6	121
MWCNTs	Hydrothermal oxidation	80	102	22	27.5	121
MWCNTs	Fenton hydroxylation	60	72	11	18.9	122
MWCNTs	NaOCl oxidation	16	104	88	543.3	123
MWCNTs	Catalytic oxidation	272	353	81	29.9	124
MWCNTs	Catalytic oxidation	272	342	70	25.8	124
SWCNTs	Acid oxidation + thermal treatment	410	770	360	87.8	125
SWCNTs	Acid oxidation + thermal treatment	381	1068	687	180.3	125
MWNTs	Air activation	65	270	205	315.4	126
MWNTs	CO_2_ activation	65	429	364	560	127
MWNTs	Chemical activation	65	785	720	1107.7	128
MWCNTs	Chemical activation	65	830	765	1180.9	128
MWCNTs	Acid oxidation	251	328	77	30.53	128
MWCNTs	Mixed-acid oxidation	251	737	485	192.9	128
MWCNTs	KOH modification	407	650	243	59.7	129
MWCNTs	Gas-phase oxidation	20	37	17	85	130
MWCNTs	Gas-phase oxidation	20	45	25	125	130
MWCNTs	Gas-phase oxidation	20	70	50	250	130
MWCNTs	Gas-phase oxidation	20	87	67	335	130

### 4.2 Advanced oxidation processes of CNTs

**Fig. 2:**
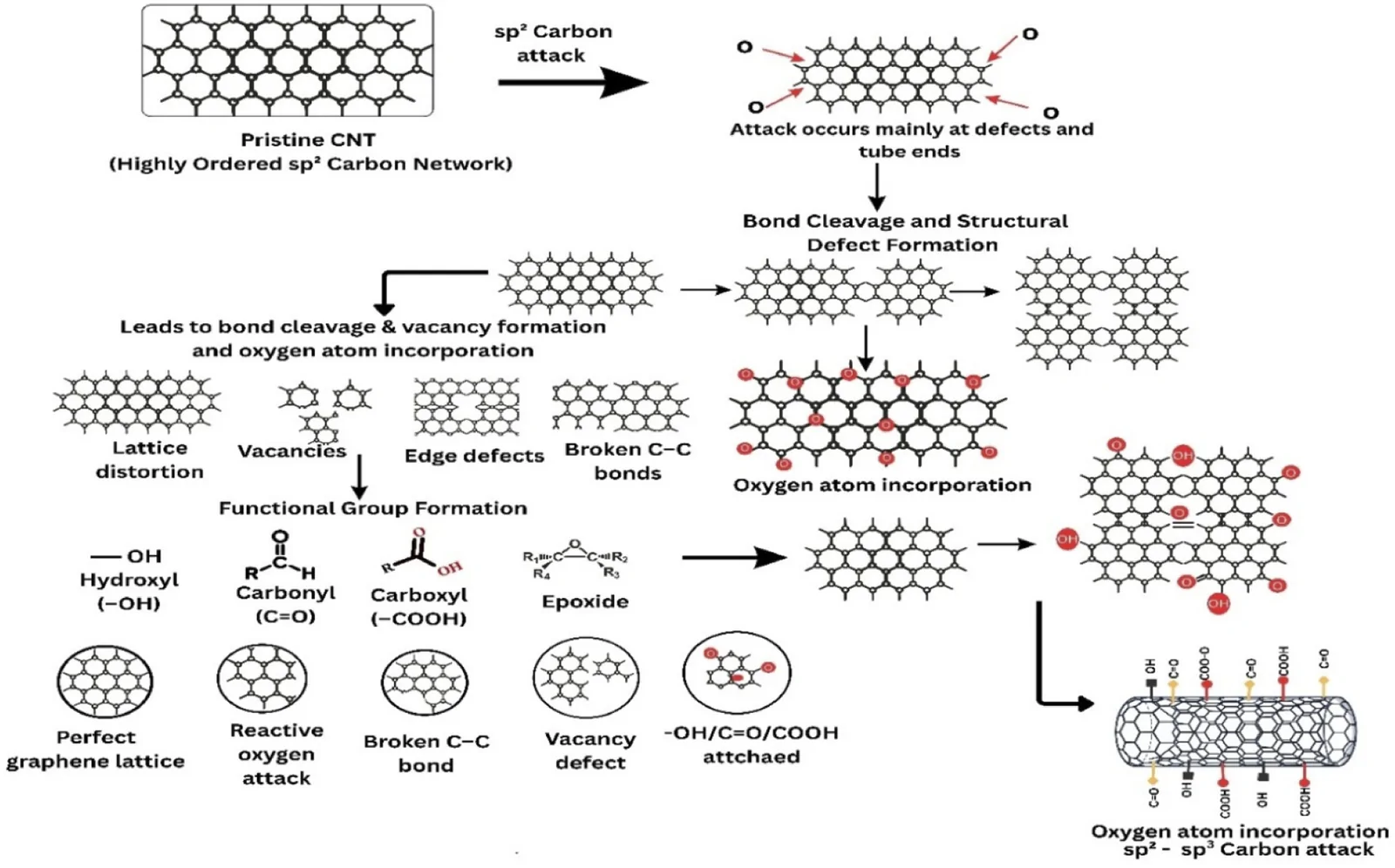
Mechanism of CNT oxidation showing defect generation and surface functionalization with oxygen-containing groups.

The oxidation process significantly changes CNT surfaces to increase the PAE adsorption sites (Fig. 2). However, the selection of the oxidation method critically determines the functional group type, density, and ultimately the PAE removal process. A comprehensive analysis of the oxidation methods shows three generalizable principles that govern CNT–PAE interactions. Oxygen content exceeding 20 wt% gradually increases PAE adsorption by 70–85% through the created surface area and hydrogen bonding capacity.^55^ The quality of functional groups, determined by their chemical structure and accessibility, is more significant than the quantity of surface area when it comes to the efficiency of PAE adsorption. The following sections critically analyse the oxidation processes, correlating processing conditions with resulting surface functionalization and their respective PAE adsorption. CNTs can be functionalized in several ways (with hydrogen peroxide, nitric acid, air/oxygen, *etc*.), and these processes introduce essential surface functional groups such as O_2_, N_2_, H, and halogens on the CNT surface. These methods include covalent, non-covalent, and defect functionalization. Surface functional groups comprising nitrogen and oxygen may increase the free valence at the exterior surface's edges due to their strong reactivity. Adding –COOH and –OH groups to the imperfect surface of pure carbon nanotubes is typically the process of defect functionalization.^56,57^ The dispersion of carbon nanotubes is enhanced by non-covalent functionalization using anionic, cationic, and non-ionic surfactants. Consequently, carbon nanotubes become more soluble and are easier to handle during processing. Another strategy for functionalization is covalent modification, which converts sp^2^ carbon atoms to sp^3^, altering the translational symmetry of the carbon nanotubes.^58,59^

#### 4.2.1 Nitric acid (HNO_3_) oxidation

The most popular technique for functionalising CNTs is nitric acid oxidation. In functionalization with aqueous solutions of HNO_3_ or a combination of H_2_SO_4_/HNO_3_, the nitrenium ion NO^2+^ attacks aromatic compounds for the introduction of surface oxides.^60,61^ It cleaves C=C bonds and introduces oxygen-containing groups, such as carboxylic acids and anhydrides, phenols, carbonyl quinones, and lactones, preferentially at highly reactive defect sites, tube ends, and sidewalls, resulting in carboxyl-dominant surface chemistry.^62,63^

1 CNT + HNO_3_ → CNT–COOH + CNT–OH + CNT–C=O + CO_2_+ NO_2_

Quantitative analysis demonstrates that MWCNTs' internal oxygen content reached 21.36 wt% when HNO_3_ and H_2_SO_4_ are used for functionalization under ultrasonication conditions.^64,65^ Their solubility in ethanol and efficacy in water treatment procedures are both enhanced by this rise in oxygen concentration. Functionalized MWCNTs demonstrate exceptional colloidal stability and enhanced dispersion property in contrast to pristine CNT flocculation. Dujardin *et al.*^66^ described a one-step process for purifying SWNTs produced by laser ablation using concentrated nitric acid. After a few minutes of sonication in concentrated nitric acid, the as-synthesised SWNTs were refluxed under magnetic stirring at 120–130 °C for four hours. The yield was between 30 and 50 wt% of the raw sample, and the metal content dropped to around 1 wt%. From the PAE adsorption perspective, nitric oxide functionalized CNTs showed optimum adsorption for polar PAE materials such as DBP and BEP. The carboxyl-dominant surface chemistry enables strong electrostatic interactions with polar PAE molecules containing ester and carbonyl functional groups. Carboxyl groups serve as critical anchoring points for covalent coupling *via* amide and ester bond formation, enabling secondary PAE-targeted modifications.^67^ However, their critical limitations must be acknowledged. Aggressive boiling conditions in the oxidation process lead to material structural loss and tube shortening. These damages lead to an increase in the diffusional resistance and sterically hinder PAE access to internal adsorption sites, adversely affecting adsorption kinetics for certain PAE compounds.

#### 4.2.2 Hydrogen peroxide oxidation

Extremely oxidizing radicals, such as O˙, H˙, and ˙OH radicals, can oxidize CNTs with minimal damage to the CNT walls (eqn (2)).^68^ The mechanism involves OH radicals with the dual characteristics of oxidizability and electrophilicity, which allow for the reaction with C=C double bonds of CNTs to create functional surface groups.^69^ Advanced oxidation processes (AOPs), including ozone/H_2_O_2_, UV/H_2_O_2_, UV/H_2_O_2_/ozone, Fenton, and photo-Fenton oxidation, are based on hydrogen peroxide as a radical precursor.^70^ Hydrogen peroxide functionalization introduces additional oxygen containing groups, thereby enhancing the dispersibility of CNTs in organic solvents. Quantitative adsorption performance data demonstrate substantial PAE binding enhancement: fulvic acid adsorption capacity increases from 109.3 mg g^−1^ for pristine CNTs to 191.2 mg g^−1^ for H_2_O_2_–UV treated samples, representing a 75% improvement.^71^ This exceeds the average 40% capacity increase observed for non-UV oxidation methods, demonstrating that photochemical activation significantly enhances functional group density and PAE binding site availability.

2 H_2_O_2_ + *hν* → 2˙OH

The H_2_O_2_–UV oxidation method offers various advantages for PAE adsorption. Minimal CNT wall damage retains structural integrity while introducing functional groups for PAE interaction. Dissolving in organic solvents is improved due to the presence of higher number of oxygenated groups, enabling homogeneous PAE–CNT contact in aqueous environmental sources. Wang *et al.*^72^ streamlined the procedure by combining two well-known reactions, the removal of metal particles with HCl and the oxidation of amorphous carbon with H_2_O_2_, into a single pot with 96% CNT purity and 50% product yield, enabling PAE adsorption onto surfaces with minimized interference from metal catalysts.^75^ H_2_O_2_ oxidation is optimal for general PAE compounds requiring balanced hydrogen bonding and electrostatic interaction mechanisms, providing versatile PAE binding across diverse PAE molecular structures.^72^

#### 4.2.3 Ozone oxidation

An economical method of creating modified carbon compounds for the removal of pollutants is ozone oxidation (O_3_). The 1,3-dipolar cycloaddition technique removes amorphous carbons from the surface of nanotubes and introduces a range of oxygenated functional groups, including alcoholic, carboxylic, ketonic, esteric, and aldehydic moieties.^73,74^ This oxidation process can occur under both aqueous and dry conditions. The solvent and reaction conditions significantly influence the specific nature and concentration of the functional groups generated. In aqueous solutions, additional hydrolytic, reductive, or oxidative workup steps further influence the outcomes of the functionalization.^75^ Quantitative PAE removal performance demonstrates exceptional efficiency: benzene removal increases from 87% for pristine CNTs to 99% for ozone-functionalized CNTs, following the Langmuir monolayer adsorption isotherm. This represents the highest pollutant removal efficiency among all the oxidation methods evaluated in this review. Ozone-induced oxidation significantly enhances CNT surface activity for PAE removal through increased surface polarity and accessible binding sites. In the aspect of PAE adsorption, this method is well suited for aromatic PAE compounds such as DBP and BEP containing two or more benzene rings.^76^

Mixed functional group composition (carboxylic + ketonic + aldehydic) preserves CNT π-system integrity while simultaneously adding hydrogen bonding capacity. This dual functionality enables both π–π interactions and hydrogen bonding (PAE–CNT), providing superior PAE binding compared to carboxyl-dominant methods. Green and economical oxidative modification is achieved through simple ozone treatment with solvent vapour, making this method cost-effective for large-scale PAE treatment applications.^77^ However, some drawbacks are present in this method, gas–solid processes make uniform oxidation challenging in macroscopic CNT agglomerations, particularly at large reaction dimensions. Bubbling ozone into aqueous CNT suspensions improves oxidation *via* the hydroxyl radical pathway (*E*° = 3.06 V, more potent than ozone *E*° = 2.06 V), though excessive oxidation may make diffusional resistance adversely affecting PAE adsorption kinetics. Overall, ozone oxidation achieves the highest PAE removal efficiency (99% benzene) while maintaining CNT structural integrity, making it the preferred method for aromatic PAE environmental remediation.^78^

#### 4.2.4 Plasma oxidation

Plasma oxidation is another effective technique for CNT surface functionalization to enhance the adsorption properties. CNTs exposed to plasma gases such as O_2_, N_2_, NH_3_, Ar, CF_4_, CO_2_, H_2_, and F_2_ develop a high surface polarity. The surfaces of CNTs experience both chemical and physical alterations due to the vigorous interactions between excited species, radicals, electrons, ions, and ultraviolet radiation in the plasma.^79,80^ It is proposed that radicals are primarily produced on the dissociated π bonds in C–C, which then interact with active oxygen atoms, because the π bonds in C–C are active and most vulnerable to plasma attack.^81^ This indicates that the C–C percentage decreased following plasma treatment. This process may result in C–O bonds, which are stabilised by the transfer of H˙ atoms from a similar or closed chain to produce C–OH bonds (eqn (3)).^82^

3 CNT + O* → CNT–C–O˙ → CNT–C–OH → O*CNT–O–C–O

A quantitative analysis of Chen *et al.*^83^ explored the possible reaction mechanism in their study on the plasma technique aimed at improving the distribution of CNTs in water. It is noted that the radical species generated during this plasma process initially form on the p bonds of C–C.^83^ The active OH radicals created by de-excitation then interact with H_2_O. Along with the O and H components produced by the plasma treatment, OH radicals that come into contact with the surface of the carbon nanoparticle eventually create C–O functional groups.^84,85^ After applying plasma, the partial oxygen content on the surfaces of multiwalled CNTs increased from 11.3 to 31.0%. After plasma treatment, the value of sp^2^ CDC atoms dropped from 53.4 to 37.2% in comparison to the CNTs that had not undergone treatment. Compared with pure CNTs, the ratios of the C–O and O–CDO functional groups increased from 4.8% and 1.9 to 16.9% and 13.7%, respectively. Compared to traditional acid oxidation methods, the aqueous stability and dispersion are greatly enhanced, ensuring reliable PAE adsorption performance in environmental water treatment applications.^86,87^

Critical analysis demonstrates that this oxidation method reaches the highest oxygen content at 31.0 wt%, providing greater PAE performance despite the moderate surface area. This observation confirms that functional group quality with optimal chemical formation is the key determinant for PAE binding efficiency, outweighing surface area quantity. Selective oxygenated group introduction enhances surface functionality without excessive structural damage, maintaining CNT integrity for sustained PAE binding. Post-treatment with atmospheric O_2_/H_2_O further enhances oxygen functional group density, improving CNT reactivity for PAE removal.^88^ However, plasma oxidation requires high equipment cost and technical complexity, limiting widespread adoption in practical environmental remediation applications. Plasma oxidation is optimal for applications requiring dual PAE and heavy metal removal, where high oxygen content (31.0 wt%) provides both hydrogen bonding sites for PAEs and electrostatic interaction sites for metal ions. The method's selective functionalization and exceptional aqueous stability make it suitable for continuous-flow PAE treatment systems despite cost limitations.

#### 4.2.5 Electrochemical oxidation

CNTs are well-suited to electrochemistry-based functionalization systems due to their high electrical conductivity. Their one-dimensional structure results in low capacitance, and the thickness of the electrochemical double layer is comparable to the nanotube diameter. A variety of experimental findings suggest that CNT surfaces exhibit rapid electron transfer across multiple redox systems, akin to the edge planes of pyrolytic graphite.^89^ The process selectively targets defect sites, open ends, and edge-plane regions, exhibiting greater reactivity than inert sp^2^-hybridized sidewalls.^90^ Mechanistically, water splitting initiates the process, forming alcohol groups on CNT surfaces that are subsequently oxidized to carboxyl and carbonyl groups. During the electrochemical functionalisation process, the anions produced in the aqueous media are driven towards the CNTs and attack the C=C bonds of aromatic rings, creating active sites for the attachment of functional groups through quinoidal structures.^91^ The two steps of electrochemical functionalisation are (i) current-enhanced intercalation of the active ions inside the nanotubes and (ii) oxidation of the nanotubes at higher voltages. The electrolyte's electrocatalytic activity depends on its nature. For instance, because OH ions can readily bind to active carbon sites, a basic electrolyte should be able to achieve a greater degree of oxidation. Protons predominate in an acid electrolyte, whereas water dissociation creates the oxidic species required for functionalisation.^92^ Surface analysis results showed that electrochemical oxidation increased the oxygen-to-carbon ratio from 0.024 to 0.038, similar to CNT–HNO_3_ functionalization. The surface functionalization displays a balanced distribution percentage of carboxyl and hydroxyl functional groups.^93^ Electrochemical tests demonstrated that CNT–EO had the highest phenol removal efficiency (0.22 ± 0.02 mg C h^−1^ cm^−2^), compared to non-oxidised CNTs (0.12 ± 0.02 mg C h^−1^ cm^−2^) and CNT–HNO_3_ (0.06 ± 0.01 mg C h^−1^ cm^−2^), with similar results for oxalate removal.^94^ The oxidation peak possible for phenol was lowest for CNT–EO (0.942 V), indicating efficient electron transfer, and its charge transfer resistance was the lowest (3.27 Ω). In addition, electrochemical oxidation enhanced the capacitive properties of MWCNTs, resulting in new redox peaks at +0.384 V and +0.441 V due to quinoid group formation. The linear relationship between the peak current and scan rate (*r* = 0.9939) confirmed a surface-controlled redox mechanism. These findings confirm that electrochemical oxidation is a simple, scalable, and effective approach to functionalize CNTs and improve their performance for applications such as supercapacitors and pollutant removal.^95^

#### 4.2.6 Thermal oxidation

Gas-phase thermal oxidation processes are widely used in CNT purification protocols but are not fully adapted for cutting, as they operate *via* a different mechanism than liquid-phase oxidation. Both approaches are known to attack structurally strained areas and defects, such as end caps and kinks. This oxidation exhibits layer-by-layer thinning of CNT shells and creates highly reactive edges. Short-term thermal oxidative exposure has shown that a single defect on the CNT surface can cause the entire graphitic wall beneath it to become gaseous.^96^ To enhance the crystalline structure of CNTs, amorphous carbon in the form of flaws or impurities is removed by sublimating it at temperatures between 400 and 800 °C. However, the prolonged oxidation process increases oxygen content, as more oxygen-containing functional groups are introduced onto the CNT surface. This modification can enhance the reactivity of the CNTs, making them more suitable for functionalization or further processing in multidisciplinary research.^97^ Thermal treatment under air was used to oxidise MWCNTs composed of benzene and ferrocene at temperatures between 270 and 600 °C. Given a minimum weight loss, the carbonyl- and hydroxyl-functionalized groups could be formed at 270–350 °C. The outcomes of the suggested functionalization approach may result in a low-cost, eco-friendly procedure.^98^

## 5 Adsorption mechanisms of CNTs on PAEs

**Fig. 3:**
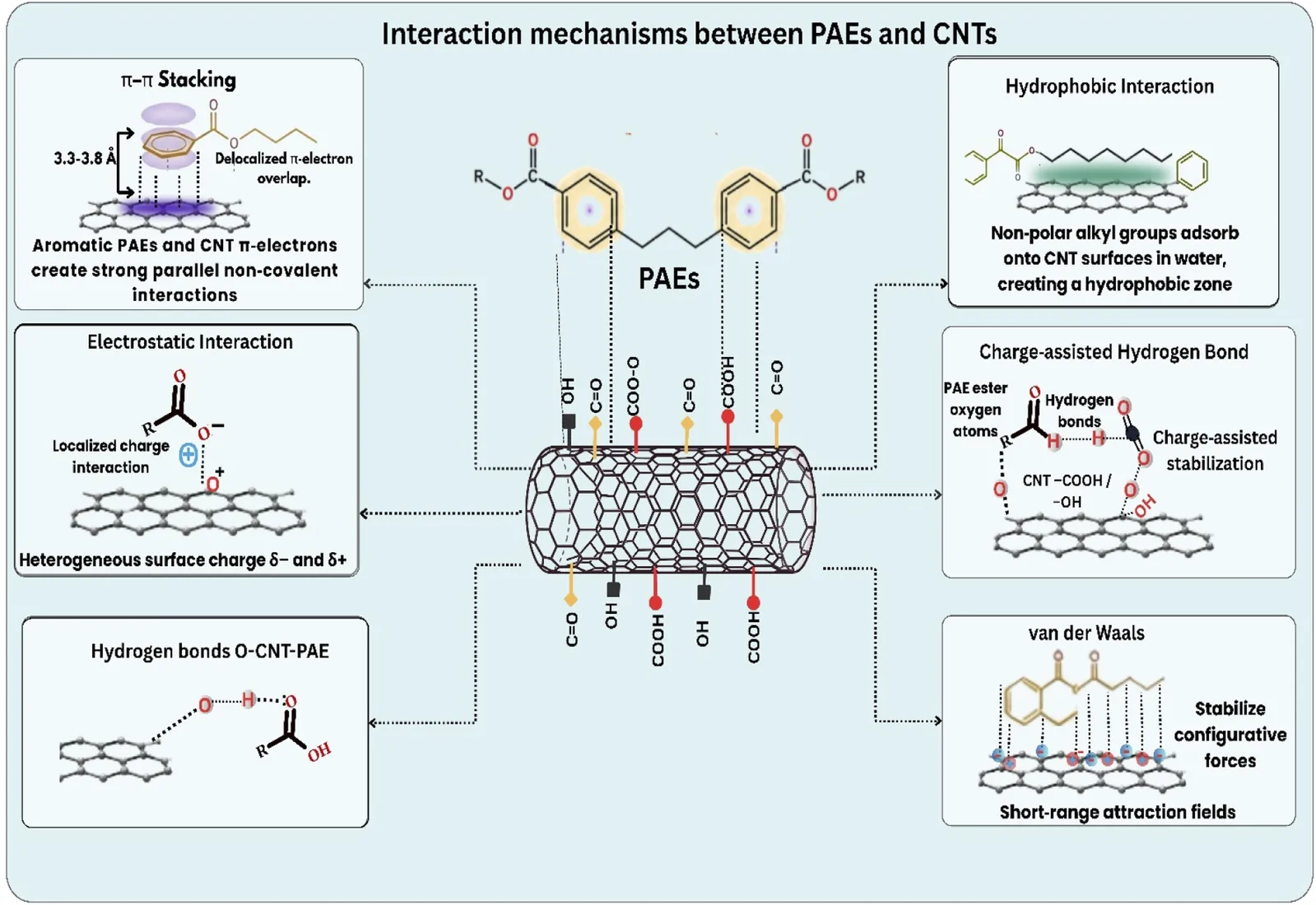
Interaction mechanisms between PAEs and CNTs.

PAEs contain significant adsorption properties due to their functional groups and aromatic structure, with benzene rings as major structural components influencing chemical behavior and molecular interactions.^99^ The selection between these mechanisms depends primarily on the PAE alkyl-chain length and CNT oxidation level. Short alkyl-chain PAEs (DMP, DBP, and DEP) exhibit increased adsorption capacity through strong π–π interactions facilitated by high π-electron density of benzene rings, whereas longer alkyl-chain PAEs (DOP and DEHP) experience steric hindrance from large carbon chains preventing effective benzene ring alignment, shifting the dominant mechanism toward hydrophobic interactions. The degree of aromaticity, substituents on aromatic rings, and solvent effects can strengthen or weaken these interactions.^100^ CNTs are naturally hydrophobic due to non-polar graphene layers, and this hydrophobic characteristic plays a significant role in adsorption of aromatic pollutants.^101^ Den *et al.*^101^ discovered that smaller MWCNTs (10–20 nm) exhibited greater adsorption capacities (8.5 mg g^−1^ for DEP) compared to activated carbon, attributed to π–π stacking interactions, while acid treatment improved the surface area and hydrophobic characteristics. Ghaffar *et al.*^103^ reported that biochar–graphene nanosheet composites treated with HNO_3_/H_2_SO_4_ considerably enhanced DMP adsorption through more polar groups increasing π–π EDA interactions. Therefore, oxidation tuning is the primary controlling factor for mechanism selection (Fig. 3) in environmental water treatment applications targeting diverse PAEs.

### 5.1 π–π stacking interaction in PAE adsorption

Benzene rings in PAEs interact with π-electron-rich CNT surfaces through π–π stacking, stabilizing adsorption with optimal ring distances around 3.3 to 4.0 Å.^102^ This mechanism involves parallel alignment of aromatic rings allowing maximum overlap of π orbitals. Critical insight is that sp^2^-hybridized carbon p-orbitals drive π–π reactivity, so excessive oxidation converting sp^2^ to sp^3^ diminishes this mechanism substantially.^103^ When CNTs are minimally oxidized with an SSA below 100 m^2^ g^−1^ and carboxyl density below 1 mmol g^−1^, π–π interactions dominate for hydrophobic aromatic PAEs achieving 60–80% removal. The π electrons of aromatic PAE rings interact with π electrons of COOH–CNTs, further enhancing adsorption as π–π stacking is stabilized by polar groups. Short alkyl-chain PAEs (DMP, DBP, and DEP) benefit more from π–π interactions due to their high π electron density and lack of steric hindrance, while longer alkyl-chain PAEs (DOP and DEHP) experience reduced π–π effectiveness due to steric hindrance from large carbon chains. Therefore, pristine or minimally oxidized CNTs are optimal for hydrophobic aromatic PAE removal through π–π stacking mechanisms.^104^

### 5.2 Role of hydrophobicity in PAE–CNT adsorption mechanisms

The hydrophobic nature of the most commonly used phthalates significantly affects their behaviour in the environment.^105^ High octanol–water partition coefficients, which estimate a material's equilibrium distribution between water and octanol and indicate its partitioning between water and sediment, are quantitative measures of a material's hydrophobic character.^106^ Strong π–π stacking interactions between hydrophobic CNTs and aromatic compounds result in irreversible adsorption. The alkyl chains in PAEs interact with CNTs *via* van der Waals and hydrophobic interactions. This kind of interaction leads to a high accumulation of PAEs on CNT surfaces, reducing their concentration in water molecules. Comparing the adsorption of various organic chemicals on a specific type of CNTs can reveal insights into different adsorption mechanisms. Comparing the adsorption properties of CNTs with varying degrees of oxidation or functionalization requires attention, as these modifications affect hydrophobicity, surface area, surface charge, and functional groups.^107^ In addition to the typical hydrophobic interactions between PAEs and activated carbon, the protonation of surface functional groups, such as amino, carboxyl, and thiol, imparts a positive charge to the surface, which can enhance electrostatic attractions. In addition to hydrophobic interactions, no other sorption mechanisms can form at neutral pH because the adsorbent and adsorbate are neutral. Adsorption is slowed down at basic pH, though, by the development of aqua-complexes due to an increase in hydroxyl ions.^108^ Diethyl phthalate is an organic compound that can exhibit hydrophobic characteristics due to its long hydrocarbon chains. This hydrophobicity can affect its solubility in water and its interaction with photocatalysts.^109^ These interactions drive PAEs to accumulate on CNT surfaces, reducing their concentration in the water phase. Functionalization of CNTs with –COOH groups improves their dispersion in water without significantly disrupting their hydrophobic core, thereby allowing better access of PAEs to adsorption sites while preserving strong π–π and hydrophobic interactions. This dual behavior, hydrophilicity at the interface and retained core hydrophobicity, makes carboxylate CNTs particularly effective for removing PAEs from aqueous systems like soft drinks and bottled beverages.

### 5.3 Role of hydrogen bonds in functionalized CNT–PAE interactions

A hydrogen bond is an intermolecular force that forms when a hydrogen atom bonds with an extremely electronegative atom, such as oxygen or nitrogen. Oxidized CNT surfaces form hydrogen bonds with ester carbonyl oxygens of PAEs, and incorporation of polyethylenepolyamine (PPA) or surface amines introduces additional H-bonding sites and, upon protonation, charge-assisted hydrogen bonds (CAHB) that further stabilize adsorption.^110^ Comparative studies (*e.g.*, benzoic acid on G-MWNTs, OH-MWNTs, COOH-MWNTs, *etc*.) demonstrate that COOH-functionalized CNTs typically exhibit higher adsorption when CAHB or strong hydrogen bonds are possible; by analogy, PAEs with accessible carbonyl groups show enhanced binding to carboxylated or amine-rich CNTs.^111,112^ Hydrogen bonds frequently act synergistically with π–π stacking and hydrophobic interactions: π–π forces align the aromatic moiety on the CNT surface, while hydrogen bonds anchor polar functional groups and reduce desorption under changing aqueous conditions. Therefore, the net adsorption behavior and selectivity of CNTs for PAEs reflect the balance of preserved hydrophobic/π interactions and introduced hydrogen-bonding or electrostatic sites determined by the type and density of surface functionalization.^113^

### 5.4 Electrostatic interactions in PAE–CNT adsorption

Electrostatic interactions, which underpin ionic bond formation and involve both attraction and repulsion, are the primary mechanism of adsorption for ionic organic molecules and ionisable organic compounds. These organic substances often gravitate towards the adsorbent surface with the opposite charge. PAEs, benzene, and graphene compounds were adsorbed onto CNTs *via* electrostatic interactions, primarily driving π–π interactions between the adsorbates. When building CNT-based sensors, it is crucial to account for electrostatic effects, as charge distribution on the CNT surface can affect interaction properties.^114^ Higher oxygen content on O-MWCNTs reduces NOM adsorption due to increased electrostatic repulsion and reduced π–π interactions. NOM sorption increases with higher ionic strength and lower pH, while stability is influenced by NOM concentration and pH, with less impact from oxygen content. Electrophoretic mobility measurements reveal that the stabilizing effects of adsorbed NOM are primarily due to steric repulsion. The interactions between functionalized MWCNTs and natural organic compounds demonstrate how surface oxygen content influences CNT stability and NOM sorption. Electrostatic interactions played a significant role in the adsorption of PAEs on carbon-based nanomaterials, affecting the overall stability and attraction of these organic complexes.^115^ These interactions arise from the attraction or repulsion between charged species, which can significantly affect the behaviour of molecules in aqueous environments. The Ewald method was used in molecular dynamics simulations to calculate electrostatic forces between PAEs and carbon-based nanomaterials (CNMs). Mulliken analysis indicated minimal charge transfer (<0.08 e), suggesting that electrostatic interactions are not the main driver of adsorption. The p*K*_a_ of benzoic acid (4.20) indicates that at natural water pH (6.5–8.5), its carboxylic group can ionise, altering the electrostatic environment and thereby affecting PAE adsorption.^116^

### 5.5 Charge-assisted hydrogen bonding in CNT–organic compound interactions

The solubility, adsorption, supramolecular association, and complexation of natural organic matter (OM) with metal ions and oxides are all impacted by weak connections between molecular segments and individual molecules. This work investigated the hypothesis that OM cohesiveness and water solubility are strongly influenced by charge-assisted hydrogen bonds (CAHB).^117^ These bonds, exemplified by structures such as (–CO_2_⋯H⋯O_2_C–), are created between weak acids that have comparable proton affinities and are noted for being shorter, more covalent, and stronger than standard hydrogen bonds. Using a high-organic reference soil, the study shows that (−)CAHBs in solid OM are disrupted by solutions of aliphatic and aromatic acids, leading to greater OM solubility.^107^ The study indicates that OM solubility increased with the concentration and molecular weight of the added organic acids, while the pH and ionic strength were held constant. In contrast, polar compounds that cannot form (−)CAHBs, such as alkanols, acetonitrile, and dimethyl sulfoxide, did not affect solubility. Under typical environmental pH conditions (5.0–10.0), both the phenolic compounds and the surfaces of carbonaceous materials tend to carry a negative charge.^109,118^ This similarity in charge leads to electrostatic repulsion, making the formation of conventional hydrogen bonds, which rely on electrostatic attraction, less favourable. Interestingly, under such conditions, a unique type of hydrogen bond known as a CAHB can still form. CAHB contributes over 42.3% to the sorption of clofibric acid (CA), acetaminophen (ACT), and *p*-aminobenzoic acid (PABA) on CNTs, while ordinary hydrogen bonds contribute less than 36.5%. The hysteresis index indicates that CAHB interactions lead to greater irreversibility in sorption. These findings emphasise the role of CAHB in enhancing the sorption of ionizable organic chemicals (IOCs) on CNTs, suggesting that materials designed with CAHB can improve the removal of IOCs from contaminated water.^119^

### 5.6 Van der Waals forces in PAE–CNT adsorption

Van der Waals forces are electrostatic forces that arise from temporarily fluctuating dipole moments due to a shift in orbital electrons, resulting in nonspecific interactions between molecules independent of chemical structure. These pressures produce short-lived bonds that become more permanent when many linkages form, inhibiting desorption, which is important for attaching high-molecular-weight organic materials to surfaces.^118^ In the context of PAE adsorption on CNTs, van der Waals forces play a dominant role through π–π stacking interactions between the aromatic benzene rings in PAEs and the graphitic π-electron system of CNTs. This π–π stacking occurs at an optimal interplanar distance of 3.4 Å. Van der Waals forces are key to understanding the adsorption of organic compounds on MWCNTs.^119,120^ Phthalates may be effectively adsorbed onto the hydrophobic surface of MWCNTs due to dispersion forces. Using 80 mg of MWCNTs, setting the sample pH to 6.0, and acetonitrile as the elution solvent are important optimisation factors for the micro-dispersive solid-phase extraction (µ-dSPE) technique. Maximum adsorption capacities (*Q*_0_) for nonpolar chemicals, such as naphthalene, varied from 42.7 mg g^−1^ (COOH-MWCNTs) to 49.0 mg g^−1^ (HO-MWCNTs), with energy parameters (*E*) of around 11.4 kJ mol^−1^, suggesting substantial dispersion forces. On the other hand, polar substances such as nitrobenzene demonstrated dipole–dipole interactions with a *Q*_0_ value of 85.1 mg g^−1^ and *E* value of 13.8 kJ mol^−1^ on HO-MWCNTs.^120^ These results indicate that van der Waals forces are a key parameter for the adsorption of organic compounds like PAE. The combination of these van der Waals interactions with hydrogen bonding and electrostatic forces enables oxidized CNTs to achieve high PAE adsorption capacities and removal efficiencies in water treatment applications.

## 6 Conclusions

PAEs are combined with polymeric materials in recent decades to increase their flexibility and durability. They are also utilised in propellants, insect repellents, and cosmetics, and their ongoing presence in waterways has posed a major threat to both the environment and human health. Oxidative functionalization of CNTs demonstrates significant potential for adsorption of PAEs in water treatment, outperforming activated carbon. This review assessed the effective oxidation methods and identified that an oxygen content exceeding 20 wt% is crucial for improved PAE adsorption. In addition, the quality of functional groups is more important than the surface area, and the molecular structure of PAE influences the selection of a suitable oxidation method. To promote the commercialization of O-CNT, several key research paths are suggested. Firstly, the optimization of O-CNT should be tailored to specific types of PAE, aligning oxidation techniques (such as HNO_3_ for polar compounds, O_3_ for aromatic structures, and electrochemical methods for mixed PAEs) to enhance adsorption levels. Following this, O-CNT composites incorporating polymer membranes, magnetic nanoparticles, and biochar should aim to lower costs and facilitate continuous-flow treatment. Additionally, pilot studies at wastewater area, groundwater sources, and drinking water plants should confirm the effectiveness of CNT absorbance with real-time data obtained over 3–6 months. Finally, future efforts should focus on optimizing oxidation conditions, scaling up production, and developing efficient recycling strategies for O-CNTs to reduce costs and facilitate their practical community applications. Certain challenges exist in the commercialization of O-CNT for PAE adsorption. The presence of co-contaminants in complex water environments, such as humic acid and heavy metals, may reduce PAE removal efficiency. Issues with reusability involve high solvent expenses, reduced effectiveness over time, and incomplete recovery of PAE. Stability concerns include the leaching of functional groups and production costs. Uncertainties regarding toxicity and gaps in regulation hinder the use of O-CNT in the treatment of drinking water.

## Author contributions

Kaviarasu G: writing – original draft preparation, review & editing, conceptualization; Selva Kumar T: writing – original draft preparation, review & editing, data curation; Raju Sasikumar: writing – review & editing, data curation, supervision; Ravikumar Rajarathinam: writing – original draft preparation, review & editing, investigation; Sheena Haorongbam: writing – original draft preparation, review & editing, data curation; Senthilkumar: writing – original draft preparation, review & editing, data curation; Amit K. Jaiswal: conceptualization, writing – review & editing, validation.

## Conflicts of interest

There are no conflicts to declare.

## Data Availability

No primary research results, software or code have been included, and no new data were generated or analysed as part of this review.
